# Accuracy of circulating microRNAs in diagnosis of sepsis: a systematic review and meta-analysis

**DOI:** 10.1186/s40560-020-00497-6

**Published:** 2020-11-02

**Authors:** Xiaomin Shen, Jiajie Zhang, Yicheng Huang, Jiepeng Tong, Li Zhang, Zhijuan Zhang, Wei Yu, Yunqing Qiu

**Affiliations:** 1 State Key Laboratory for Diagnosis and Treatment of Infectious Diseases, National Clinical Research Center for Infectious Diseases, Collaborative Innovation Center for Diagnosis and Treatment of Infectious Diseases, Zhejiang Provincial Key Laboratory for Drug Clinical Research and Evaluation, The First Affiliated Hospital, Zhejiang University School of Medicine, Hangzhou, China; 2 Department of Infectious Diseases, Zhejiang Provincial People’s Hospital, People’s Hospital of Hangzhou Medical College, Hangzhou, China

**Keywords:** Sepsis, SIRS, miRNAs, Diagnosis, Biomarkers

## Abstract

**Objectives:**

The aim of this study was to systematically assess the accuracy of circulating microRNAs (miRNAs) as a promising biomarker for sepsis via a meta-analysis.

**Methods:**

PubMed, Cochrane Library, Embase, Web of Science, Scopus, and Ovid databases were searched up to April 3, 2020. The Quality in Prognostic Studies (QUADAS-2) tool was used to assess methodological quality. The pooled sensitivity (Sen), specificity (Spe), positive or negative likelihood ratios (PLR or NLR), diagnostic odds ratio (DOR), curve, and area under the curve (AUC) were calculated with 95% confidence interval (95% CI). The overall accuracy (OA) of miRNAs, procalcitonin (PCT), and C-reactive protein (CRP) was analyzed by the chi-square test.

**Results:**

A total of 22 records were eligible for systematic review, including 2210 sepsis, 426 systemic inflammatory response syndrome (SIRS), and 1076 healthy controls (HC). The pooled Sen, Spe, and DOR of miRNAs were 0.80 (95% CI 0.75–0.83), 0.85 (95% CI 0.80–0.89), and 22 (15–32), respectively. The DOR of PCT and CRP were 17 (95% CI 4–68) and 7 (95% CI 1–48), respectively. The OA value of miRNAs (79.02%) and PCT (76.95%) were higher than CRP (61.22%) (*P* < 0.000). The subgroup analysis indicated that miRNAs in adults, serum type, downregulation of miRNA expression, criteria of Sepsis-3, internal reference of non-U6, and dysregulation expression of miR-223 had superior diagnostic accuracy. In addition, there was no significant publication bias among the included studies. Fagan’s nomogram showed valuable clinical utility.

**Conclusions:**

Our meta-analysis indicated that the level of circulating miRNAs, particularly the miR-223, could be used as an indicator for sepsis.

**Supplementary Information:**

**Supplementary information** accompanies this paper at 10.1186/s40560-020-00497-6.

## Introduction

Sepsis is defined as a life-threatening condition of organ dysfunction resulted from a dysregulated host response to infection [1]. Recent epidemiologic data have shown an increasing incidence of sepsis and septic shock with high mortality [2]. Although recent data suggested a declining trend in mortality, longer-term morbidity and decreased health-related quality of life remain a serious problem [3]. Additionally, the clinical syndrome of sepsis is difficult to diagnose. A previous study reported 50% of patients with sepsis were not correctly classified in the USA [4]. Early diagnosis of sepsis is crucial for improving the survival rate; however, traditional screening tools and biomarkers lack specificity [5].

MicroRNAs (miRNAs) are small non-coding RNAs, associated with the presence and severity of sepsis. Dysregulation of several miRNAs, such as miR-146a, miR-223, miR-15a, miR-16, and miR-150, was found in the peripheral blood of sepsis patients [6–9]. Previous studies have demonstrated the expression of miRNAs in sepsis had specific compartment characteristics, and serum-derived exosome miRNAs were considered to be a significant diagnosis marker and survival prediction factor [10]. In addition, miRNAs had shown to be involved in the regulation of the exacerbated inflammation, endothelial dysfunction, and coagulation cascade in sepsis [11]. miR-15a, miR-125b, and miR-146a have been shown to prevent NF-κB activation in sepsis by repressing TRAF6 and IRAK expression [10, 11]. However, the expression level of miRNAs showed differences in sepsis due to different population characteristics and normalization methodologies among different studies [11]. Therefore, a comprehensive and systematic meta-analysis of currently available data will facilitate the understanding of the diagnostic accuracy of these miRNAs in sepsis.

## Methods

### Search strategy and selection criteria

This meta-analysis was presented according to the Preferred Reporting Items for Systematic Reviews and Meta-Analyses statement (PRISMA) [12]. Six databases including PubMed, Cochrane Library, Embase, Web of Science, Scopus, and Ovid were searched by the end of April 3, 2020. The retrieval terms included “sepsis” or “Severe Sepsis” or “Pyemia” or “Septicemia” and “micro RNA” and “microRNAs” or “miRNA.”

Literatures were considered eligible for inclusion using the following criteria: (1) the target population consisted of one or more circulating miRNAs for sepsis; (2) sufficient data to generate true positive (Tp), true negative (Tn), false positive (Fp), and false negative (Fn) directly or indirectly; and (3) either retrospective or prospective design studies. Studies that are excluded in our meta-analysis met the following criteria: (1) reviews, letters, correspondence, expert opinions, and editorial; (2) animal or in vitro studies; (3) duplicated information; and (4) insufficient information regarding the diagnosis value.

### Data collection

Two investigators (XMS and WY) independently screened the records retrieved from the search after deduplication by title and abstract. Full-text studies that satisfied all the inclusion criteria were further screened for eligibility by the same investigators. Any discrepancies were resolved by a third reviewer (JJZ). Data extraction was performed by two review authors (JJZ and YCH) independently, including author and year of publication, sample characteristics, diagnostic criteria, illness severity (APACHE II and SOFA score), and numbers of Tp, Tn, Fp, and Fn observations.

### Assessment of methodologic quality

Quality Diagnostic Accuracy Studies-2 (QUADAS-2) was used to assess the methodological qualities of the included studies by independent authors (XMS and WY) [13]. Disagreements were resolved by a third reviewer (JPT).

### Data synthesis and statistical analysis

The threshold effect was evaluated before data synthesis. The diagnostic index tests, including sensitivity (Sen), specificity (Spe), positive or negative likelihood ratios (PLR or NLR), diagnostic odds ratio (DOR), summary receiver operating characteristic (SROC), curve, and area under the curve (AUC), were quantified with 95% confidence interval (95% CI). Meta-analysis was performed using a bivariate random-effects model to estimate the summary diagnostic indexes. Heterogeneity was explored using meta-regression models, and subgroup analyses were further analyzed according to varied factors. Publication bias was evaluated by Deeks’ funnel plot, and clinical utility was evaluated by Fagan’s nomogram. STATA version 14 (STATA Corp, College Station, TX, USA) was used for all statistical analyses. The differences between the overall accuracy (OA), that is the proportion of Tp and Tn in all evaluated cases, of miRNAs, procalcitonin (PCT), and C-reactive protein (CRP) were analyzed by the chi-square test using SPSS Statistics 22 (IBM, China).

## Results

### Search results and methodological qualities of included studies

**Fig. 1:**
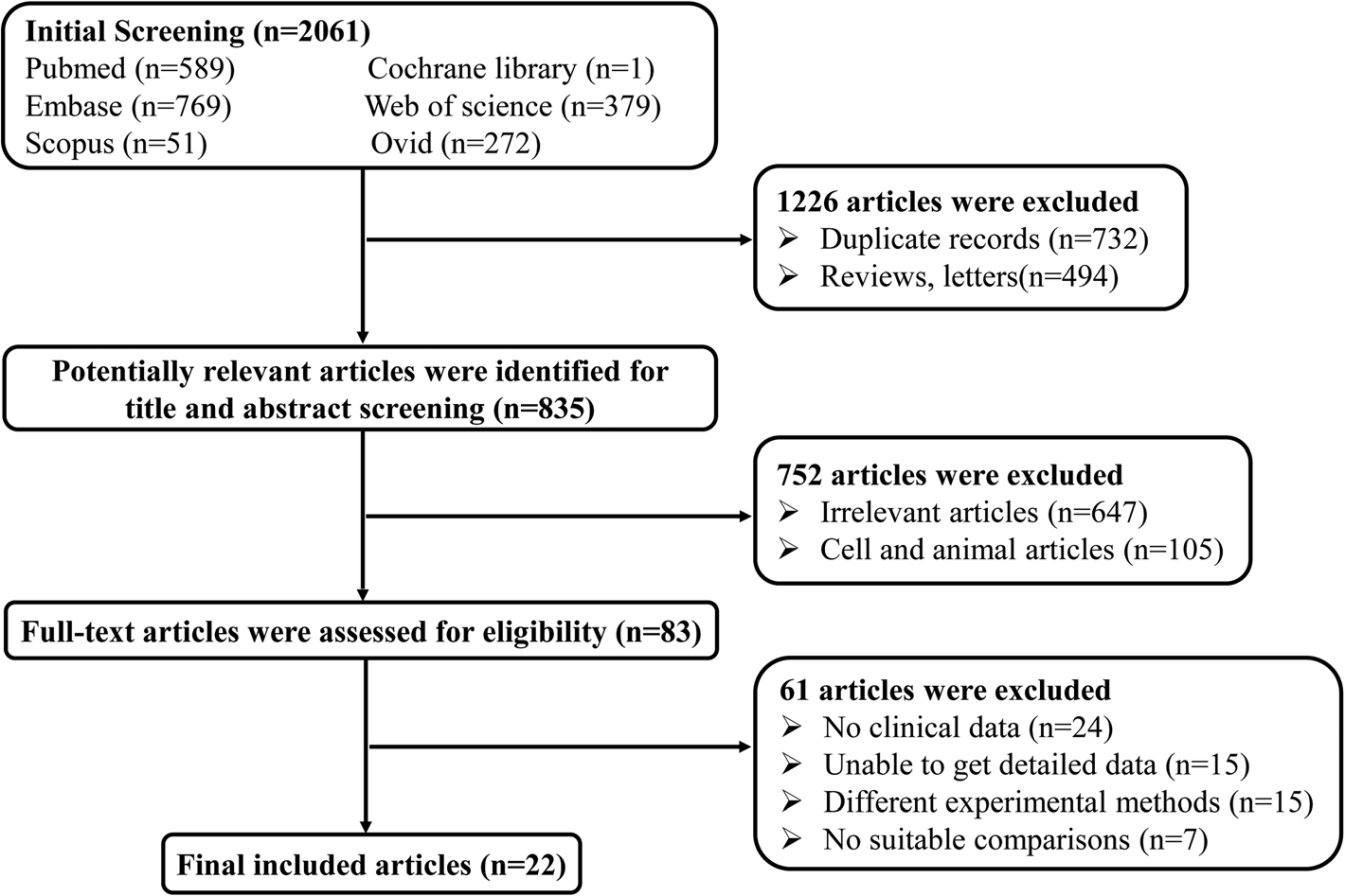
Flow diagram of the study selection

Our search yielded 2061 references through 6 electronic databases. After removing 1978 duplicates, animal or in vitro studies, irrelevant articles, and excluded article format, a total of 83 full-text references were screened for eligibility. Finally, 22 records were included in our systematic review [6–9, 14–31]. The detailed flow diagram of the study selection process is shown in Fig. 1.

The methodological qualities of the included articles were evaluated according to the QUADAS-2 criteria (Supplementary Figure 1). Risk of bias is mainly derived from the index test. The conduct or interpretation of the index test introduced high risk in 6 records [7, 8, 17, 19, 23, 28] and unclear risk in 13 articles [6, 14–16, 18, 20, 21, 25–27, 29–31].

### Characteristics of included studies

A total of 2337 patients, including 14 studies of systemic inflammatory response syndrome (SIRS), 2 studies of local infections, and 14 studies of healthy controls (HC), provided data regarding the diagnostic accuracy of miRNAs. The characteristics of the included studies and patients are presented in Table 1 and Supplemental Table 1. Single miRNAs were reported in all studies. Among the 29 studies, 8 studies were about the expression of miRNAs in children, and 21 studies were about adults. In addition, there were 15 studies of serum specimens and 13 studies of plasma specimens.

**Table 1:** Characteristics of the included studies and participants

First author	Year	Population	Region	miRNAs	Regulation mode	Specimen	DC	Internal reference types in qRT-PCR	Sample size				Diagnostic power			Ref.
									Case	Number	Control	Number	Sen (%)	Spe (%)	AUC	
Wang JF	2010	Adults	China	miR-146a	Downregulated	Serum	1	mmu-miR-295	Sepsis	50	SIRS	30	63.3	100	0.804	[6]
Wang JF	2010	Adults	China	miR-223	Downregulated	Serum	1	mmu-miR-295	Sepsis	50	SIRS	30	80.0	100	0.858	[6]
Wang H	2012	Adults	China	miR-15a	Downregulated	Serum	1	U6	Sepsis	166	SIRS	32	68.3	94.4	0.858	[7]
MA Y	2013	Adults	UK	miR-150	Upregulated	Whole blood	2	None	Sepsis	23	SIRS	22	72.7	85.7	0.830	[8]
MA Y	2013	Adults	UK	miR-4772-5p-iso	Downregulated	Whole blood	2	None	Sepsis	23	SIRS	22	68.2	71.4	0.760	[8]
Wang L	2013	NR	China	miR-146a	Downregulated	Plasm	NR	U6	Sepsis	14	SIRS	14	60.0	87.5	0.813	[9]
Wu YH	2013	Children	China	miR-223	Upregulated	Plasm	2	cel-miR-39	Sepsis	35	SIRS	20	82.9	85.0	0.919	[14]
Wu YH	2014	Children	China	miR-146a	Upregulated	Plasm	2	cel-miR-39	Sepsis	40	SIRS	20	77.5	77.0	0.815	[15]
Wu YH	2014	Children	China	miR-223	Upregulated	Plasm	2	cel-miR-39	Sepsis	40	SIRS	20	77.5	55.0	0.678	[15]
Liu CL	2015	Children	China	miR-223	Upregulated	Plasm	2	None	Sepsis	49	HC	50	83.5	86.0	0.904	[16]
Yang YL	2015	Adults	China	miR-499	Upregulated	Plasm	2	None	Sepsis	112	HC	20	86.7	90.8	0.838	[17]
Han Y	2016	Adults	China	miR-143	Upregulated	Serum	1	U6	Sepsis	103	SIRS	95	78.6	91.6	0.910	[18]
Lan C	2016	Adults	China	miR-155-5p	Upregulated	Serum	3	None	Sepsis	105	HC	35	85.3	80.6	0.855	[19]
Lan C	2016	Adults	China	miR-133a-3p	Upregulated	Serum	3	None	Sepsis	105	HC	35	97.9	54.8	0.769	[19]
Xu J	2017	Adults	China	miR-195	Upregulated	Serum	1	U6	Sepsis	54	SIRS	41	75.6	70.4	0.776	[20]
Rahmel T	2018	Adults	German	miR-122	Upregulated	Serum	3	cel-miR-54	Sepsis	108	SIRS	20	58.3	95.0	0.760	[21]
Wu XL	2018	Adults	China	miR-223	Upregulated	Plasm	1	U6	Sepsis	187	HC	186	56.6	86.6	0.754	[22]
Abou El-Khier	2018	Adults	Egypt	miR-122	Upregulated	Serum	NR	cel-miR-39	Sepsis	25	LI	25	100	100	1.000	[23]
Guo HL	2019	Adults	China	miR-495	Downregulated	Serum	2	U6	Sepsis	105	HC	100	89.5	83.0	0.915	[24]
Karam RA	2019	Children	Egypt	miR-146a	Downregulated	Serum	2	U6	Sepsis	55	HC	60	86.6	56.6	0.803	[25]
Zhang WP	2019	Adults	China	miR-7110-5p	Upregulated	Plasm	3	miR-16	Sepsis	44	HC	21	84.1	90.5	0.833	[26]
Zhang WP	2019	Adults	China	miR-223-3p	Upregulated	Plasm	3	miR-16	Sepsis	44	HC	21	82.9	100	0.964	[26]
Lin YJ	2019	Adults	China	miR-210	Upregulated	Plasm	1	None	Sepsis	125	HC	110	81.0	80.9	0.852	[27]
Lin YJ	2019	Adults	China	miR-494	Upregulated	Plasm	1	None	Sepsis	125	HC	110	80.9	72.1	0.847	[27]
Lin YJ	2019	Adults	China	miR-205	Downregulated	Plasm	1	None	Sepsis	125	HC	110	78.6	90.5	0.86	[27]
Zhu XP	2019	Adults	China	miR-125b	Upregulated	Plasm	2	U6	Sepsis	120	HC	120	49.2	80.0	0.658	[28]
Liu GZ	2020	Children	China	miR-181a	Downregulated	Serum	2	U6	Sepsis	102	LI	50	83.3	84.0	0.893	[29]
Rabab F	2020	Children	Egypt	miR-101	Upregulated	Serum	2	U6	Sepsis	50	SIRS	30	84.0	84.0	0.908	[30]
Rabab F	2020	Children	Egypt	miR-187	Upregulated	Serum	2	U6	Sepsis	50	SIRS	30	72.0	76.0	0.789	[30]
Xu HM	2020	Adults	China	miR-19b-3p	Downregulated	Serum	2	U6	Sepsis	103	HC	98	85.4	85.7	0.921	[31]

*qRT-PCR* Quantitative RT-PCR, *DC* Diagnostic criteria, *SIRS* Systemic inflammatory response syndrome, *HC* Healthy controls, *LI* Local infections, *Sen* Sensitivity, *Spe* Specificity, *AUC* Area under the curve, *Ref.* References, *NR* No report

### Threshold effect

The Spearman correlation coefficient for miRNAs was 0.127 (*P* = 0.503) using a rank correlation test. The shape of the ROC plot was not arm and shoulder shape (Supplementary Figure 2a). These results indicated the heterogeneity among the included studies was not caused by the threshold effect [32].

### Diagnostic accuracy of miRNAs, procalcitonin, and C-reactive protein for sepsis

The random-effects model was used to synthesize the data due to significant heterogeneity among the 30 studies (*I*^2^ = 86.49% for Sen, *I*^2^ = 78.03% for Spe, *I*^2^ = 100% for DOR). Outliers, falling outside 95% CI, could be the main source of heterogeneity [Supplementary Figure 2b].

**Fig. 2:**
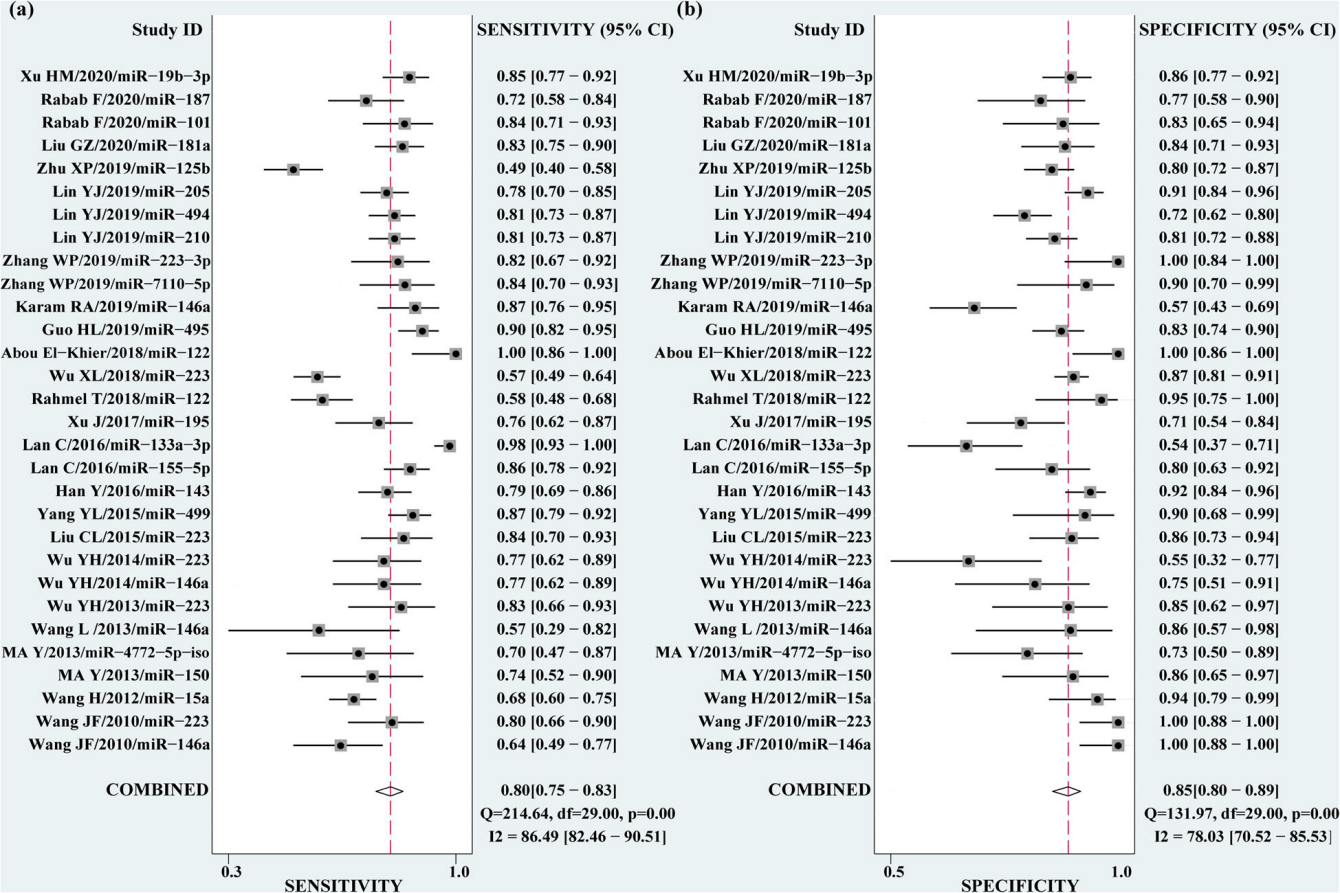
Forest plots of pooled sensitivity (Sen) and specificity (Spe) of circulating miRNAs for the diagnosis of sepsis. **a** Sen. **b** Spe

**Fig. 3:**
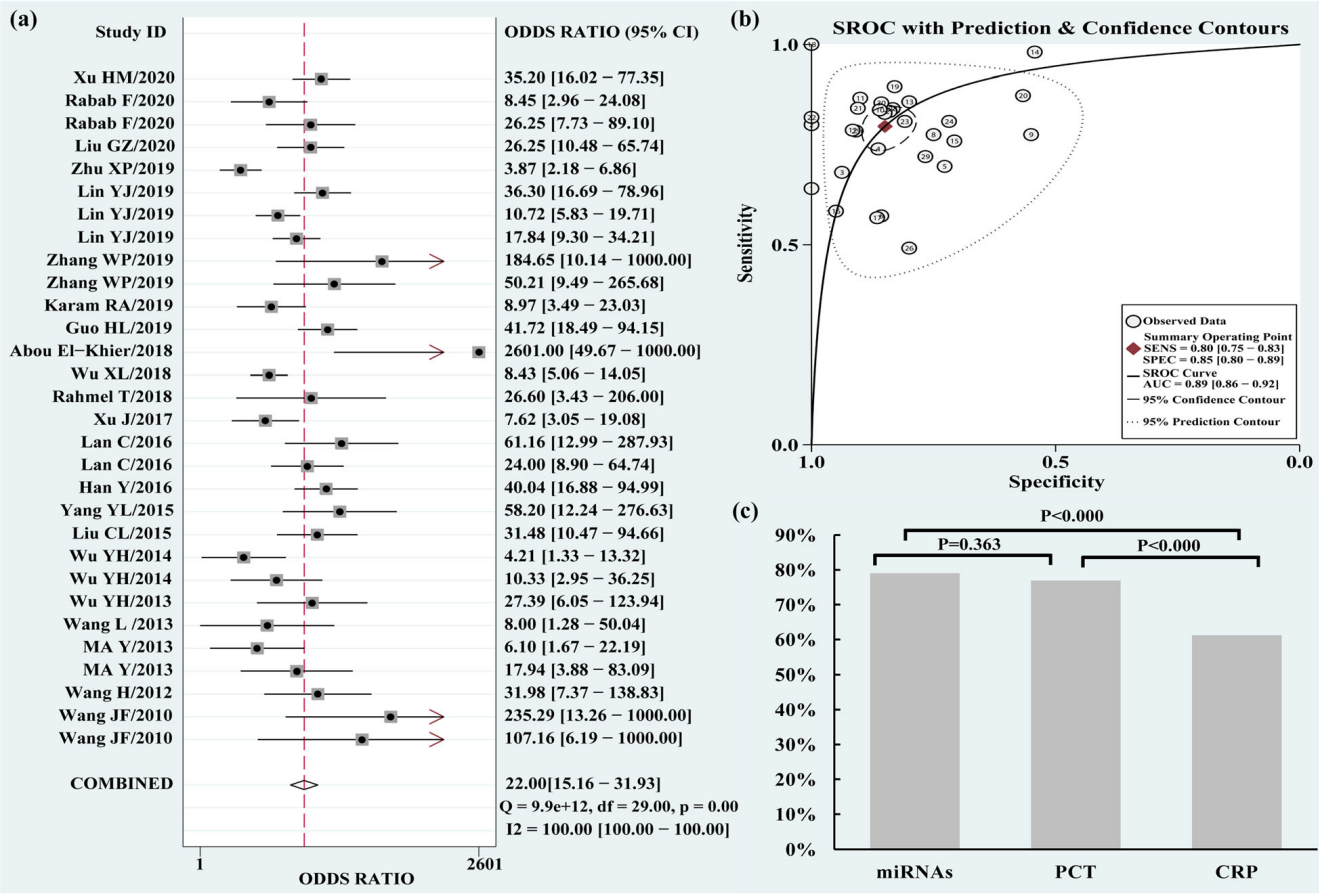
Diagnostic odds ratio (DOR), summary receiver operating characteristic (SROC) curve, and overall accuracy (OA) value of circulating miRNAs for the diagnosis of sepsis. **a** DOR. **b** SROC curve. **c** OA value

The pooled Sen and Spe were 0.80 (95% CI 0.75–0.83) and 0.85 (95% CI 0.80–0.89), respectively (Fig. 2). The PLR and NLR were 5.3 (95% CI 4.0–6.9) and 0.24 (95% CI 0.20–0.29), respectively. The DOR of pooled studies was 22 (15–32), and the AUC for SROC was 0.89 (95% CI 0.86–0.92), indicating a high overall accuracy of circulating miRNAs for sepsis (Table 2 and Fig. 3a, b).

**Table 2:** Diagnostic accuracy of miRNAs, PCT, and CRP for sepsis

Index	Number of studies	Number of patients	Sen (95% CI)	Spe (95% CI)	PLR (95% CI)	NLR (95% CI)	DOR (95% CI)	AUC (95% CI)	OA (%)
miRNAs	30	3914	0.80 (0.75–0.83)	0.85 (0.80–0.89)	5.3 (4.0–6.9)	0.24 (0.20–0.29)	22 (15–32)	0.89 (0.86–0.92)	79.02
PCT	4	347	0.73 (0.66–0.79)	0.86 (0.61–0.96)	5.2 (1.6–16.8)	0.31 (0.23–0.43)	17 (4–68)	0.74 (0.70–0.77)	76.95
CPR	4	245	0.77 (0.73–0.81)	0.71 (0.29–0.94)	2.6 (0.7–9.4)	0.37 (0.18–0.73)	7 (1–48)	0.77 (0.73–0.81)	61.22
miR-223	6	732	0.77 (0.67–0.84)	0.91 (0.73–0.97)	8.3 (2.5–27.9)	0.25 (0.17–0.38)	33 (8–142)	0.87 (0.84–0.90)	79.25

*miRNAs* micrornas, *PCT* Procalcitonin, *CRP* C-reactive protein, *95% CI* 95% confidence intervals, *Sen* Sensitivity, *Spe* Specificity, *PLR* Positive likelihood ratios, *NLR* Negative likelihood ratios, *DOR* Diagnostic odds ratio, *AUC* Area under the curve, *OA* Overall accuracy

In all of these studies, 4 studies analyzed the diagnostic accuracy of PCT for sepsis; the pooled Sen and Spe were 0.73 (95% CI 0.66–0.79) and 0.86 (95% CI 0.61–0.96), respectively. The PLR and NLR were 5.2 (95% CI 1.6–16.8) and 0.31 (95% CI 0.23–0.43), respectively. The DOR of pooled studies was 17 (95% CI 4–68), and the AUC for SROC was 0.74 (95% CI 0.70–0.77) (Table 2).

Furthermore, 4 studies analyzed the diagnostic accuracy of CRP for sepsis; the pooled Sen and Spe were 0.77 (95% CI 0.73–0.81) and 0.71 (95% CI 0.29–0.94), respectively. The DOR of pooled studies was 7 (95% CI 1–48), and the AUC for SROC was 0.77 (95% CI 0.73–0.81) (Table 2).

The OA value of miRNAs (79.02%) and PCT (76.95%) were higher than CRP (61.22%) (*P* < 0.000). The OA value confirmed no significant difference in the diagnostic accuracy between miRNAs and PCT (*P* = 0.373) (Fig. 3c).

### Diagnostic accuracy of miR-223

miR-223 was reported in 6 studies of collected researches. The pooled Sen, Spe, AUC, and DOR were 0.77 (95% CI 0.67–0.84), 0.91 (95% CI 0.73–0.97), 0.87 (95% CI 0.84–0.90), and 33 (95% CI 8–142), respectively (Table 2).

### Meta-regression and subgroup analysis

**Fig. 4:**
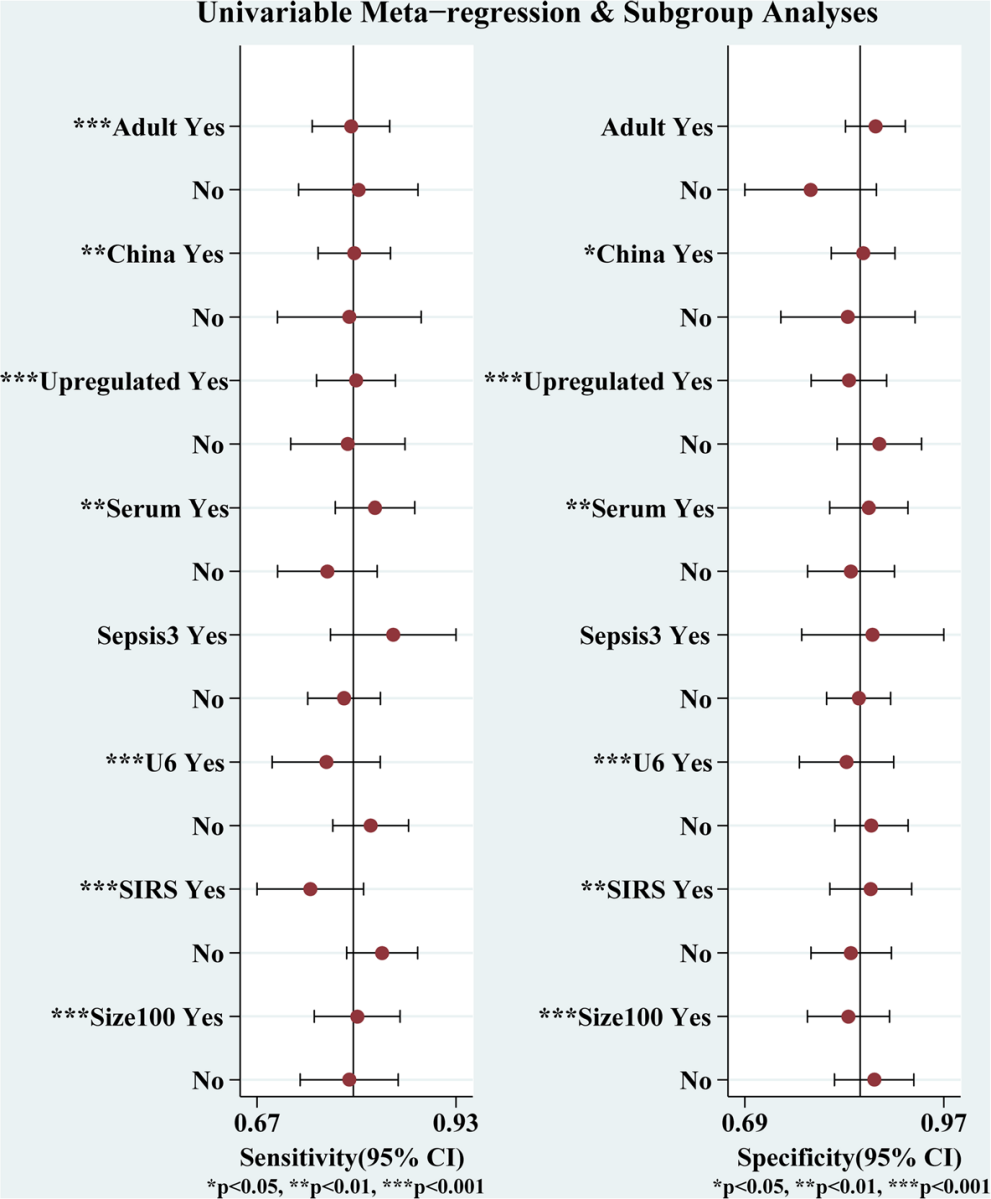
Meta-regression and subgroup analysis

The heterogeneity was explored using multiple univariable bivariate meta-regression models. The effect of each covariate on Sen was estimated separately from that on Spe (Fig. 4). The diagnostic criteria did not have an effect on miRNA Sen and Spe (*P* > 0.05), and the covariate of the population did not have an effect on Spe. In addition, the rest of the covariates were statistically significant for Sen and Spe.

Subgroup analyses of miRNAs were further performed due to obvious heterogeneity. As shown in Table 3, among studies examining only adult patients, the Sen and Spe of circulating miRNAs were 0.80 (0.73–0.85) and 0.88 (0.82–0.92), respectively. The DOR of studies using serum samples was higher than that of studies using plasma samples (32 versus 17). The Sen, Spe, and DOR of miRNAs in patients with sepsis reference to Sepsis-3 were higher than those in patients reference to Sepsis-1 or Sepsis-2. Non-U6, as an internal control to normalize the expression levels of miRNAs, showed superiority compared with U6. The pooled Sen, Spe, PLR, NLR, DOR, and AUC in the non-U6 group were 0.79 (0.70–0.86), 0.96 (0.82–0.99), 19.5 (3.9–97.8), 0.22 (0.15–0.32), 89 (15–518), and 0.90 (0.87–0.92), respectively. In the subgroup of control groups, the pooled Sen and Spe of miRNAs for sepsis were 0.74 (0.69–0.78) and 0.87 (0.79–0.92) versus SIRS and 0.83 (0.76–0.88) and 0.82 (0.76–0.87) versus HC, respectively.

**Table 3:** Summary diagnostic power based on subgroup analyses

Subgroups	Number of studies	Cases number of sepsis	Cases number of controls	Sen (95% CI)	Spe (95% CI)	PLR (95% CI)	NLR (95% CI)	DOR (95% CI)	AUC (95% CI)
Population									
Adults	21	1902	1283	0.80 (0.73–0.85)	0.88 (0.82–0.92)	6.4 (4.4–9.4)	6.4 (4.4–9.4)	28 (17–46)	0.91 (0.88–0.93)
Children	8	421	280	0.81 (0.77–0.85)	0.77 (0.67–0.84)	3.47 (2.4–5.0)	0.24 (0.19–0.30)	14 (8–24)	0.82 (0.79–0.86)
Region									
China	23	2003	1368	0.80 (0.75–0.84)	0.85 (0.80–0.89)	5.4 (4.0–7.1)	0.24 (0.19–0.30)	23 (15–33)	0.89 (0.86–0.92)
Non-China	7	334	209	0.80 (0.67–0.89)	0.84 (0.69–0.93)	5.1 (2.4–11.0)	0.24 (0.13–0.43)	21 (7–71)	0.89 (0.86–0.91)
Regulation mode									
Upregulated	20	1544	1031	0.80 (0.74–0.85)	0.83 (0.78–0.87)	4.8 (3.5–6.4)	0.24 (0.18–0.32)	20 (13–32)	0.89 (0.86–0.91)
Downregulated	10	793	546	0.80 (0.73–0.85)	0.89 (0.80–0.94)	7.3 (4.1–13.1)	0.23 (0.18–0.29)	32 (18–56)	0.89 (0.86–0.91)
Specimen									
Serum	15	1231	711	0.82 (0.76–0.88)	0.87 (0.79–0.93)	6.4 (3.9–10.5)	0.20 (0.15–0.27)	32 (19–54)	0.91 (0.88–0.93)
Plasm	13	1060	822	0.77 (0.70–0.82)	0.84 (0.78–0.88)	4.7 (3.4–6.6)	0.28 (0.21–0.37)	17 (10–29)	0.88 (0.84–0.90)
Diagnostic criteria									
Sepsis-3	5	406	132	0.85 (0.69–0.94)	0.87 (0.69–0.95)	6.8 (2.8–16.1)	0.17 (0.08–0.35)	40 (20–81)	0.93 (0.90–0.95)
Sepsis-1/2	23	1892	1406	0.78 (0.73–0.82)	0.84 (0.79–0.88)	4.8 (3.7–6.2)	0.26 (0.22–0.32)	18 (13–26)	0.87 (0.84–0.90)
Internal reference types									
U6	12	1109	856	0.76 (0.68–0.83)	0.83 (0.77–0.87)	4.4 (3.3–5.8)	0.29 (0.21–0.39)	15 (9–24)	0.87 (0.84–0.89)
Non-U6	9	436	207	0.79 (0.70–0.86)	0.96 (0.82–0.99)	19.5 (3.9–97.8)	0.22 (0.15–0.32)	89 (15–518)	0.90 (0.87–0.92)
Control groups									
SIRS	14	806	426	0.74 (0.69–0.78)	0.87 (0.79–0.92)	5.7 (3.5–9.4)	0.30 (0.26–0.36)	19 (11–33)	0.82 (0.78–0.85)
HC	14	1404	1076	0.83 (0.76–0.88)	0.82 (0.76–0.87)	4.6 (3.4–6.1)	0.21 (0.15–0.30)	22 (14–35)	0.89 (0.86–0.91)
Sample size									
> 100	15	1746	1181	0.81 (0.73–0.87)	0.83 (0.77–0.87)	4.7 (3.6–6.2)	0.23 (0.17–0.33)	20 (13–30)	0.89 (0.86–0.91)
< 100	15	591	396	0.78 (0.74–0.82)	0.88 (0.79–0.94)	6.7 (3.7–12.2)	0.24 (0.20–0.30)	27 (13–58)	0.85 (0.82–0.88)

*miRNAs* MicroRNAs, *95% CI* 95% confidence intervals, *Sen* Sensitivity, *Spe* Specificity, *PLR* Positive likelihood ratios, *NLR* Negative likelihood ratios, *DOR* Diagnostic odds ratio, *AUC* Area under the curve, *SIRS* Systemic inflammatory response syndrome, *HC* Healthy controls

### Publication bias

A funnel graph was performed to assess the likelihood of publication bias. Deeks’ test indicated no potential publication bias in all the variables analyzed in this study (Supplementary Figure 2c).

### Clinical utility of index test

Fagan’s nomogram was used to assess the post-test probabilities. When the pre-test probability was set at 20%, the post-test probability arrived at 57% with PLR of 5 and 6% with NLR of 0.24 (Supplementary Figure 2d).

## Discussion

Sepsis is a significant public health problem, with high mortality and long-term morbidity [3]. Good evidence of a mortality benefit in the early diagnosis and treatment of sepsis and septic shock [5]. However, it is not always easy to distinguish sepsis and SIRS in the early stage and is even sometimes impossible. A number of studies have investigated miRNAs as efficient biomarkers in various phases of sepsis [11]. Therefore, the present study comprehensively evaluated the diagnostic accuracy of circulating miRNAs in sepsis patients via a systematic review and meta-analysis.

In this study, we found the Sen, Spe, and DOR of circulating miRNAs for diagnosis of sepsis were higher than PCT and CRP. The OA value of miRNAs and PCT were significantly higher than CRP, while no significant difference was found between miRNAs and CRP. The results of the subgroup analysis showed adults and patients diagnosed by reference to Sepsis-3 had a higher value of DOR. In addition, improvement of miRNA accuracy for the detection of sepsis was observed in the subgroup of serum type, internal reference of non-U6, and downregulation of miRNA expression.

Although different screening tools and biomarkers, such as white cell count, neutrophil count, interleukin 6 (IL-6), CRP, and PCT, have been used for sepsis diagnosis, none of them is proven to be specific [5]. Previous studies demonstrated CRP lack specificity for sepsis due to an increase in non-infectious inflammatory conditions [33]. The levels of PCT could help to guide the antibiotic therapy in patients with sepsis, while the heterogeneous data of PCT failed to provide guidance for the benefits of sepsis patients [34, 35]. Current literature suggested miRNAs played a vital role in the pathophysiology of sepsis and could distinguish the various phases of sepsis [6–9, 11, 14–31]. Several miRNAs, such as miR-146-a, miR-125b, and miR-223, were positively or negatively associated with sepsis severity [22, 25, 28]. In this present study, the AUC of miRNAs was 0.89 (0.86–0.92), which is higher than that of PCT [0.74 (0.70–0.77)] and CRP [0.77 (0.73–0.81)]. However, the OA value showed no significant difference between miRNA and PCT. Notably, a number of miRNAs have proved to be not only as diagnostic markers (miR-15a, miR-16, miR-223, miR-499-5p) but also as prognostic markers (miR-193b, miR-483-5p, and miR-574-5p) for sepsis [6–9, 36]. These results indicated circulating miRNAs could have the ability to monitor the progress of sepsis.

Despite the efficacy of miRNAs, there was obvious heterogeneity in this meta-analysis. Patients diagnosed by reference to Sepsis-3 had higher Sen, Spe, and DOR of miRNAs than that of patients reference to Sepsis-1 or Sepsis-2. As a deeper understanding of the biology of sepsis, the criteria of Sepsis-3 introduced a framework based on susceptibility, pathogen, dysregulated host response, and organ dysfunction [35]. The different specimen types could have impacted the conflicting results. Our results found the accuracy of miRNAs for sepsis diagnosis in the serum samples was higher than that in the plasma. Contrastingly, other disease studies reported the expression of miRNAs in the plasma was higher than the serum due to more proteins in the plasma [37]. The same may be true of internal reference types. However, there has been no unified standard for the normalization of miRNA. Thus, multicenter standardization researches need to be explored for better elucidation of miRNAs as a promising biomarker in sepsis.

Our results showed dysregulation expression of miR-223 showed a superior diagnostic achievement. miR-223, as a pro-inflammatory factor, is involved in several signaling pathways that control inflammatory responses and infection reaction activation, such as negative regulation of STAT3 and IL-6 expression during sepsis [38–40]. Wu et al. [22] showed miRNA-223 expression was positively correlated with APACHE II score as well. Of note, Wang et al. [6] suggested that miR-223 in serum was downregulated among sepsis patients, while other five studies demonstrated miR-223 in the plasm was upregulated [14–16, 22, 26]. In fact, the conflicting results of miR-223 were also reported in hepatitis and hepatocellular carcinoma [39]. The possible explanations were that the expression of miR-223 was different among different sepsis stages and different experimental methods. However, the sample sizes for the evaluation of miR-233 were not large enough. Therefore, more additional researches are needed to assess the benefit of miR-233 during the course of sepsis.

Despite miRNAs as a promising diagnostic biomarker for sepsis, there are still several potential limitations. First, heterogeneity was observed in our meta-analysis. However, there was no significant threshold effect. Further subgroup analyses improved the heterogeneity. Second, miRNAs in the included patients were confirmed after sepsis diagnosis by the clinic criteria, which may add population selection bias. In addition, the included studies did not evaluate the correlation between miRNA expression and illness severity in detail. Furthermore, we could not control the statistical methods among the included studies, which may influence the results of the meta-analysis.

## Conclusions

In conclusion, circulating miRNAs, especially for miR-223, are potential markers for distinguishing sepsis from SIRS and HC. Better results could be obtained for adults and patients diagnosed by reference to Sepsis-3. Further large and well-designed studies should be explored to identify the role of promising miRNAs in sepsis.

## Supplementary Information

**Additional file 1: Supplementary Figure 1.** A summary of methodological qualities included articles using the QUADAS-2 criteria.

**Additional file 2: Supplementary Figure 2.** The ROC plot, Galbraith, Deeks’ funnel plot, and Fagan’s Nomogram of the diagnostic meta-analysis. (a) ROC plot; (b) Galbraith; (c) Deeks’ funnel plot; (d) Fagan’s Nomogram.

**Additional file 3: Supplemental Table 1**. The research type and severity characteristics of 22 included studies

## Data Availability

All data generated or analyzed during this study are included in this published article and its supplementary information files.

## Abbreviations

miRNAs: MicroRNAs
QUADAS-2: Quality in Prognostic Studies
Sen: Sensitivity
Spe: Specificity
PLR: Positive likelihood ratios
NLR: Negative likelihood ratios
DOR: Diagnostic odds ratio
AUC: Area under the curve
OA: Overall accuracy
SROC: Summary receiver operating characteristic
95% CI: 95% confidence intervals
PCT: Procalcitonin
CRP: C-reactive protein
SIRS: Systemic inflammatory response syndrome
HC: Healthy controls
IL-6: Interleukin 6
