# Supplementary figures and images for: Accuracy of circulating microRNAs in diagnosis of sepsis: a systematic review and meta-analysis
Source: J Intensive Care. 2020 Nov 2;8:84. doi: 10.1186/s40560-020-00497-6 (PMC7607638; doi:10.1186/s40560-020-00497-6)

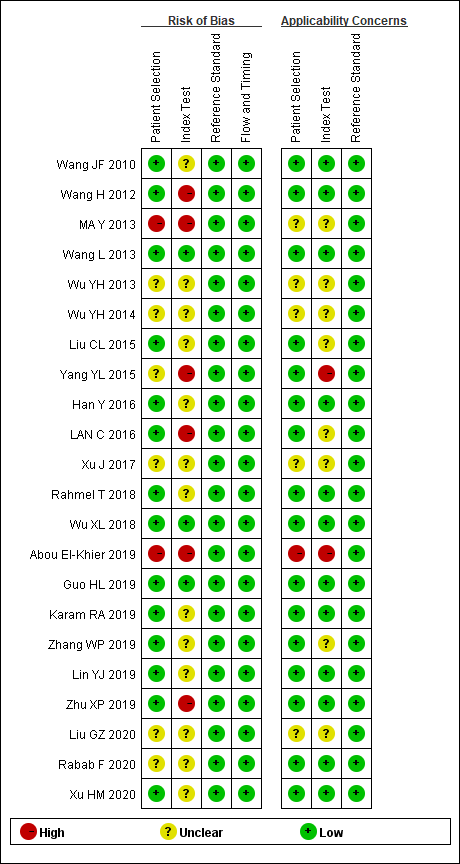

Supplement: Supplementary file 1 — Additional file 1: Supplementary Figure 1. A summary of methodological qualities included articles using the QUADAS-2 criteria. [file 40560_2020_497_MOESM1_ESM.tif]
