# Supplementary material for: Accuracy of circulating microRNAs in diagnosis of sepsis: a systematic review and meta-analysis
Source: J Intensive Care. 2020 Nov 2;8:84. doi: 10.1186/s40560-020-00497-6 (PMC7607638; doi:10.1186/s40560-020-00497-6)
Supplement: Supplementary file 3 — Additional file 3: Supplemental Table 1. The research type and severity characteristics of 22 included studies [file 40560_2020_497_MOESM3_ESM.docx]

**Supplemental Table 1** The research type and severity characteristics of 22 included studies

| First author | Research type | Time of blood sampling | APACHE II score（Mean/IQR or ± SD） | SOFA (Mean/IQR or ± SD） | Ref. |
| --- | --- | --- | --- | --- | --- |
| Wang JF | Retrospectively | within 24 | 15.7±6.3 | NR | [6] |
| Wang H | Cross-sectional | within 24 | 15.75±6.46 | NR | [7] |
| MA Y | Retrospectively | NR | 18 (9–27) | 3 (1–8) | [8] |
| Wang L | Prospectively | NR | NR | NR | [9] |
| Wu YH | Retrospectively | within 12 | NR | NR | [14] |
| Wu YH | Retrospectively | within 12 | NR | NR | [15] |
| Liu CL | Retrospectively | within 12 | NR | NR | [16] |
| Yang YL | Retrospectively | within 24 | 23.45±8.03 | NR | [17] |
| Han Y | Retrospectively | NR | 15.9± 6.2 | NR | [18] |
| Lan C | Prospectively | NR | Sepsis 11.74±5.11 Severe sepsis 13.17±6.61 Septic shock 16.74±7.61 | 5.03±4.01 5.85±3.41 7.81±4.21 | [19] |
| Xu J | Prospectively | within 12 | NR | NR | [20] |
| Rahmel T | Prospectively | within 24 | NR | 11.0±3.8 | [21] |
| Wu XL | Prospectively | within 24 | 16 (12–20) | NR | [22] |
| Abou El-Khier | Prospectively | NR | NR | NR | [23] |
| Guo HL | Prospectively | within 24 | 12.92±3.22 | 5.28±1.31 | [24] |
| Karam RA | Prospectively | within 24 | NR | NR | [25] |
| Zhang WP | Prospectively | NR | NR | 6.47 ± 3.11 | [26] |
| Lin YJ | Prospectively | NR | 29.46±3.42 | 13.15±2.23 | [27] |
| Zhu XP | Retrospectively | within 24 | 15.4 ± 6.4 | 7.2 ± 3.5 | [28] |
| Liu GZ | Retrospectively | NR | NR | NR | [29] |
| Rabab F | Prospectively | NR | NR | NR | [30] |
| Xu HM | Prospectively | within 24 | 13.50 (11.00) | 5.74±1.41 | [31] |

IQR, interquartile range;
